# Gaze instability after exposure to moving visual stimuli in patients with persistent postural-perceptual dizziness

**DOI:** 10.3389/fnhum.2022.1056556

**Published:** 2022-11-25

**Authors:** Chihiro Yagi, Yuka Morita, Tatsuya Yamagishi, Shinsuke Ohshima, Shuji Izumi, Kuniyuki Takahashi, Kosuke Itoh, Yuji Suzuki, Hironaka Igarashi, Arata Horii

**Affiliations:** ^1^Department of Otolaryngology Head and Neck Surgery, Graduate School of Medical and Dental Sciences, Niigata University, Niigata, Japan; ^2^Center for Integrated Human Brain Science, Brain Research Institute, Niigata University, Niigata, Japan

**Keywords:** chronic dizziness, persistent postural-perceptual dizziness, gaze instability, eyetracking test, visual stimuli

## Abstract

**Introduction:**

Persistent postural-perceptual dizziness (PPPD) is a chronic vestibular syndrome lasting more than 3 months. The core vestibular symptoms are dizziness, unsteadiness, and non-spinning vertigo, which are exacerbated by upright posture or walking, active or passive motion, and exposure to moving or complex visual stimuli. Among these, visual exacerbation is a key feature of PPPD for which the neural mechanisms are unknown. We hypothesized that vestibular symptoms may be exacerbated by visual stimuli through gaze behavioral change after exposure to moving or complex visual stimuli. The study aimed to examine gaze stability after exposure to moving visual stimuli in patients with PPPD.

**Methods:**

Fourteen healthy controls (HCs), 27 patients with PPPD, and 12 patients with unilateral vestibular hypofunction (UVH), showing chronic vestibular symptoms for >3 months, were enrolled in the study. The participants were instructed to fixate on the gazing point at the center of a screen for 30 s before and after 90 s of exposure to moving visual stimuli. Gaze stability, best represented by the bivariate contour ellipse area (BCEA), was compared among three groups, both before and after exposure to the moving visual stimuli. Comparisons between pre- and post-moving visual stimuli in BCEA were also conducted. Correlation between the post/pre ratio of BCEA and vestibular tests, several clinical symptom scales including the Dizziness Handicap Inventory, Niigata PPPD Questionnaire, and Hospital Anxiety and Depression Scale, and the exacerbation of dizziness by exposure to moving visual stimuli was examined in the PPPD group.

**Results:**

BCEA, both before and after exposure to moving visual stimuli in the PPPD group, was not different from that in HC and UVH groups. In the PPPD group, BCEA increased significantly after exposure to moving visual stimuli. The post/pre ratio of BCEA correlated with the occurrence of exacerbation of the dizziness sensation by exposure to moving visual stimuli; however, it did not correlate with vestibular tests or clinical symptom scales.

**Conclusion:**

Patients with PPPD were more likely to exhibit gaze instability after exposure to moving visual stimuli, which potentially exacerbated vestibular symptoms. This phenomenon may help elucidate the neural mechanisms of visual exacerbation in patients with PPPD.

## Introduction

Persistent postural-perceptual dizziness (PPPD), which has been included in the 11th revision of the International Classification of Diseases, is a persistent chronic vestibular syndrome characterized by vestibular symptoms lasting longer than 3 months typically preceded by acute vestibular disorders (Staab et al., 2017). PPPD has three exacerbating factors: upright posture or walking, active or passive motion, and exposure to moving or complex visual stimuli. Among these, it has been demonstrated that visual stimuli have the broadest area under the receiver operating characteristic curve for diagnosing PPPD (Yagi et al., 2019), and the visual stimulation-dominant subtype is the most common subtype of PPPD revealed by cluster analysis (Yagi et al., 2021), suggesting that visual exacerbation is a key feature of PPPD for which the neural mechanisms are unknown. Gazing stably at a single point plays an important role in suppressing motion perception in the visual field (Martinez-Conde et al., 2004). Previous reports have indicated that dizziness and postural instability induced by visual stimuli are more likely to develop in adults who have difficulty maintaining a stable gaze (Winkler and Ciuffreda, 2009; Ombergen et al., 2016). Chaudhary et al. (2022) investigated the characteristics of gaze stability and movement of the center of pressure using a series of conditions with increasing levels of complexity and concluded that adults with visually induced dizziness exhibit gaze instability and increased postural and head sway compared with healthy adults. PPPD is known to persist for a prolonged period once visual exacerbation occurs (Staab et al., 2017); however, to date, gaze stability in PPPD following visual stimulation has not been reported. Therefore, we considered it clinically valuable to confirm if a change of gaze stability occurs after visual stimulation. We hypothesized that vestibular symptoms may be exacerbated by visual stimuli through changes in gaze stability on a stationary target after exposure to moving or complex visual stimuli. To verify this hypothesis, gaze stability before and after exposure to moving visual stimuli was measured in patients with PPPD, healthy controls (HCs), and patients with chronic unilateral vestibular hypofunction (UVH). In addition, the relationship between changes in gaze stability and clinical symptom scales, psychiatric status, results of vestibular tests, and exacerbation of the dizziness sensation by exposure to moving visual stimuli was examined in patients with PPPD.

## Materials and methods

### Patients

This study included 14 HCs, 27 patients with PPPD, and 12 patients with UVH who were diagnosed at the Department of Otolaryngology Head and Neck Surgery at Niigata University Medical and Dental Hospital. Patients who presented themselves at the hospital from January 2021 to March 2022 and an unselected sample of consecutive patients with PPPD or UVH who visited our department during that period were recruited. Approximately one-third of the patients recruited eventually agreed to participate and were included in the study. For the HC group, participants were selected and recruited from the hospital staff to match the age and sex of the PPPD group.

All participants had normal eyesight, either naked eye or corrected visual acuity. The HCs reported no history of balance disorders and had normal neurological function. PPPD was diagnosed using the Barany Society criteria (Staab et al., 2017). Patients with UVH had chronic vestibular symptoms lasting more than 3 months and unilateral abnormal values in caloric testing, according to a report by Starkov et al. (2021). The precipitating conditions for patients with PPPD or UVH are shown in Supplementary Table 1. Among the patients with PPPD or UVH, two patients with PPPD were diagnosed with an anxiety disorder at the first visit to our department and received psychiatric treatment. No other psychiatric comorbidities were observed. In the PPPD group, three patients had unilateral low-tone sensorineural hearing loss, two had migraine, and one had tinnitus. In the UVH group, four patients had unilateral sensorineural hearing loss, three had bilateral sensorineural hearing loss, and two had hypertension. None of the patients with PPPD had spontaneous, positional, or head-shaking nystagmus. In the UVH group, six patients demonstrated head-shaking nystagmus, two of whom also had spontaneous nystagmus. All patients with UVH had head motion-induced dizziness; however, they were asymptomatic when stationary. They had neither visually induced dizziness nor persistent dizziness. At the time of the gaze stability test, 13 patients with PPPD had been taking antidepressants, such as selective serotonin reuptake inhibitors and serotonin and noradrenaline reuptake inhibitors for more than 3 months. The other patients with PPPD and UVH had vestibular rehabilitation as treatment. None of the patients with UVH were taking antidepressants.

### Eye tracking and visual stimuli presentation

Eye movements were recorded by a commercially available screen-based eye tracker (Tobii Pro Nano, Tobii Technology K.K., Tokyo, Japan) with a sampling rate of 60 Hz. The stimuli presented on the G-Tune H5 laptop screen (MouseComputer Co., Tokyo, Japan) with a pixel resolution of 1,920 × 1,080 (34.5 × 19.4 cm) were generated using After Effects software (Adobe Inc., San Jose, CA, United States). The screen was located approximately 65 cm in front of the participant, who sat on a steady chair in a dimly lit room. All measurements were performed with the participant’s head stabilized using a chin rest to minimize head movements. The details of the Tobii I-VT algorithm are shown in Supplementary Table 2.

**FIGURE 1:**
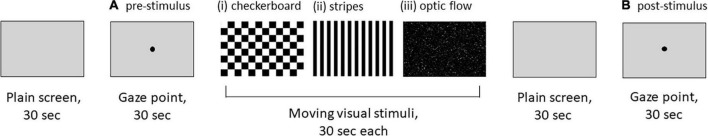
An order of visual stimuli for the gaze stability test. (i) Checkerboard pattern stimulus comprising 8 rows × 12 columns of squares reversed in contrast (100%) at 12 Hz. (ii) Optokinetic stimulus with 12 black-and-white vertical stripes sweeping across a screen at 6 s. (iii) Radial optic flow stimulus with moving white dots (size: 0.1∼1.1 degrees of visual angle, speed: 3 s with a flat speed gradient) on a black background expanding from the center of the screen. **(A,B)** A stationary gazing black dot (5 mm diameter fixation target subtending approximately 0.4 of visual angle) in the center of the light gray screen [RGB (211, 211, 211)].

The order of the stimuli in the gaze stability test is shown in Figure 1. Moving visual stimuli, which consisted of (i) a checkerboard pattern stimulus comprising 8 rows × 12 columns of squares reversed in contrast (100%) at 12 Hz, (ii) optokinetic stimulus by 12 black-and-white vertical stripes sweeping across a screen at 6 s, and (iii) radial optic flow stimulus with moving white dots (size: 0.1∼1.1 degrees of visual angle, speed: 3 s with a flat speed gradient) on a black background expanding from the center of the screen, were continuously presented on a PC screen for 30 s each. These moving visual stimuli were created in an attempt to reproduce stimuli that are likely to exacerbate symptoms in patients with PPPD in daily life, such as (i) the flashing lights on a television, (ii) scenery flowing sideways when viewed from the inside of a train, and (iii) scenery flowing from front to back when sitting in the passenger seat of a car. A stationary gazing black dot (5 mm diameter fixation target subtending approximately 0.4 of the visual angle) in the center of the light gray screen [RGB (211, 211, 211)], which the participant was asked to stare at, was presented for 30 s before (A: pre-stimulus) and after (B: post-stimulus) the moving visual stimuli. At the beginning of the gaze stability test and immediately after the presentation of the moving visual stimuli, a light gray plain screen for truncation was presented for 30 s each.

The recorded data were filtered through the Tobii I-VT algorithm available in the analysis software Tobii Pro Lab (Tobii Technology K.K., Tokyo, Japan) (Tobii, 2022), with reference to a previous study (Tsitsi et al., 2021). The I-VT filter calculates whether a sequence of gaze sample belongs to the same fixation or is part of a saccade, using a velocity criterion. The default value of 30 s was used for the velocity threshold, a setting that allows for relatively short and fast movements to be detected as saccades. A gaze was counted as one fixation if there was a gaze pause of 60 ms or more. If the gaze position shifted by more than 0.5 of the visual angle, it was counted as another fixation. In summary, if the gaze moved slowly, at a speed of less than 30 s, the number of fixations would increase, but it would not be counted as a saccade.

### Measurement of gaze stability

Participants were instructed to always keep their eyes on the center of the screen during the gaze stability test, regardless of whether they were looking at moving visual stimuli, a stationary gazing black dot, or a light gray plain screen. The following five parameters of gaze stability were measured before, during, and after exposure to moving visual stimuli: number of fixations (count/30 s), mean duration of fixation (s), number of saccades (count/30 s), standard deviation (SD) of the horizontal/vertical gaze position (degrees of visual angle), and bivariate contour ellipse area (BCEA) (square degrees of visual angle). The BCEA, a mathematical description of gaze stability (Steinman et al., 1982), was defined using the following equation:


$$B\,C\,E\,A=\pi \,\chi^{2}\,\sigma_{x}\,\sigma_{y}\,\sqrt{\left(1-\rho^{2}\right),}$$

where χ^2^ is the chi-square value (two degrees of freedom) corresponding to a probability value of 0.682 (i.e., ± 1 SD), σx and σy correspond to the SDs of the horizontal and vertical gaze positions, respectively, and correspondsρ to the Pearson correlation coefficient between the horizontal and vertical gaze positions. The BCEA provides the area of the ellipse that encompasses 68% of the gaze positions within a trial. Therefore, larger BCEA values indicate lower gaze stability.

### Clinical symptom scale

#### Dizziness handicap inventory

The Dizziness handicap inventory (DHI) is a standard 25-question questionnaire designed to quantitatively evaluate the degree of handicap felt by patients with vestibular disorders in their daily lives (Jacobson and Newman, 1990, Goto et al., 2011). The total score ranges from 0 to 100, with 0 indicating no disability and 100 indicating severe disability.

#### Hospital anxiety and depression scale

The Hospital anxiety and depression scale (HADS) is a questionnaire consisting of self-administered anxiety and depression subscales. Each HADS subscale was assessed with seven questions (Zigmond and Snaith, 1983). Each question was scored on a scale of 0 (not at all) to 3 (most of the time, very often). Therefore, the total score for each HADS subscale was 21, and the full HADS score was 42, with higher scores indicating higher levels of anxiety and depression.

#### The Niigata persistent postural-perceptual dizziness questionnaire

The Niigata PPPD Questionnaire (NPQ) is a self-administered questionnaire used to perform screening and assess the severity of PPPD (Yagi et al., 2019). The NPQ consists of 12 questions that assess the degree of exacerbation of symptoms for three exacerbating factors: upright posture or walking, active or passive movement, and visual stimulation. The severity of each factor was evaluated using four questions, with a score from 0 (none) to 6 (unbearable) for each question. Thus, the total score for each factor was 24, and the full score for the NPQ was 72, with higher scores indicating greater severity.

### Vestibular tests

#### Posturography

The patients underwent static posturography on a solid or rubber foam surface using Gravicoda^®^ (ANIMA Corp., Tokyo, Japan) with open and closed eyes. The elliptical balance area (cm^2^), adopted in previous studies as a representative index of the degree of postural sway, was used as an indicator for testing on a solid surface. The foam ratio (posturography with/without foam) during the eyes-closed condition was used as an index of somatosensory dependence of postural control, whereas the Romberg ratio on the foam was used as an index of visual dependence (Okumura et al., 2015).

#### Bithermal caloric testing

Bithermal caloric testing was performed by stimulating each external auditory canal twice with air at 26^°^C and 45^°^C for 60 s at 5 min intervals. The maximum slow phase velocity was measured using an electronystagmography and canal paresis (CP) (%) and calculated using Jongkee’s index formula (Jongkees et al., 1962). A CP value of 25% or more was considered to indicate significant unilateral caloric weakness.

#### Exacerbation of dizziness sensation after the gaze stability test

After the gaze stability test, participants were interviewed regarding any exacerbation of the dizziness sensation by exposure to moving visual stimuli. The interviews were conducted at least within 5 min after the end of the test and were judged positive if there was an exacerbation of the dizziness sensation and negative if there was not.

### Statistical analyses

To compare the clinical and demographic characteristics of HCs, PPPD, and UVH, chi-square tests were performed for sex differences, and the Kruskal−Wallis test followed by the *post-hoc* Dann−Bonferroni test was performed for age, elliptical balance area, Romberg ratio on foam, foam ratio during an eyes-closed condition, and HADS. The Mann–Whitney U test was performed for the duration of disease, CP, DHI, and NPQ in the PPPD and UVH groups, both of which had dizziness symptoms.

The repeated measures two-way analysis of variance (ANOVA) followed by the *post-hoc* Sidak and Tukey test was performed on the parameters of gaze stability in the stationary gazing black dot screen before (A: pre-stimulus) and after (B: post-stimulus) stimulation to compare the changes before and after moving visual stimuli and the differences among the three groups. The repeated measures two-way ANOVA followed by the *post-hoc* Tukey test was also performed to evaluate the differences in the parameters of gaze stability among the three types of moving visual stimuli.

Finally, to identify the clinical correlates of gaze stability in PPPD, the post/pre BCEA ratio (post-stimulus BCEA divided by pre-stimulus BCEA on the stationary gazing black dot screen) was compared between those with and without antidepressant medication and exacerbation of the dizziness sensation by moving visual stimuli using the Mann–Whitney U test, and correlation of the post/pre BCEA ratio was tested with clinical symptom scales (DHI, NPQ, and HADS) and results of vestibular tests using the Spearman’s correlation coefficient.

All statistical analyses were performed using Graph Pad Prism version 9 (GraphPad Software, San Diego, CA, United States). Statistical significance was set at *p* < 0.05. The effect size for *r* of 0.10, 0.30, and 0.50 was considered small, medium, and large, respectively.

## Results

### Clinical and demographic characteristics of the healthy control, persistent postural-perceptual dizziness, and unilateral vestibular hypofunction groups

**FIGURE 2:**
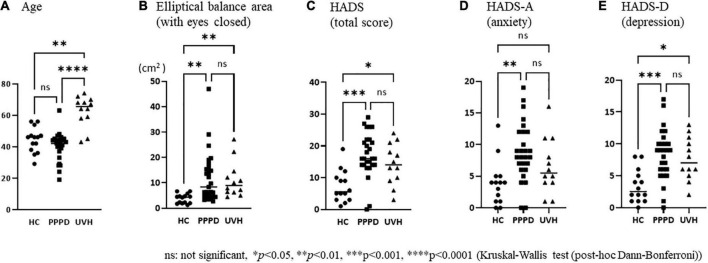
Comparisons of the clinical and demographical characteristics among the three groups. **(A)** The mean age of patients in the unilateral vestibular hypofunction (UVH) group [median: 65.5 years, interquartile range (IQR): 11.3 years] was significantly higher than that of the participants in the healthy control (HC) (median: 46 years, IQR: 11.3 years) (Dunn’s test, *p* < 0.01, *r* = 0.60) and persistent postural-perceptual dizziness (PPPD) (median: 42 years, IQR: 8.0 years) (Dunn’s test, *p* < 0.0001, *r* = 0.74) groups. **(B)** The elliptical balance area (with eyes closed) of the HC group (median: 4.36, IQR: 3.01) was significantly smaller than that of the PPPD (median: 8.39, IQR: 11.6, *p* = 0.001, *r* = 0.55) and UVH (median: 8.97, IQR: 7.66, *p* = 0.002, *r* = 0.66) groups. **(C)** The Hospital Anxiety and Depression Scale (HADS) (total score) of the HC group (median: 5.5, IQR: 7.5) was significantly lower than that of the PPPD (median: 16.0, IQR: 8.0, *p* < 0.001, *r* = 0.64) and UVH (median: 14.0, IQR: 11.0, *p* = 0.046, *r* = 0.48) groups. **(D)** The HADS-A (anxiety) of the HC group (median: 4.0, IQR: 4.0) was significantly lower than that of the PPPD group (median: 8.0, IQR: 6.0, *p* < 0.01, *r* = 0.54). **(E)** The HADS-D (depression) of the HC group (median: 2.5, IQR: 4.3) was significantly lower than that of the PPPD (median: 9.0, IQR: 4.0, *p* < 0.001, *r* = 0.60) and UVH (median: 7.0, IQR: 5.5, *p* < 0.05, *r* = 0.46) groups.

There were no significant differences in sex among the three groups (Table 1). The mean age of patients in the UVH group [median: 65.5 years, interquartile range (IQR): 11.3 years] was significantly higher than that of the participants in the HC (median: 46.0 years, IQR: 11.3 years) (Dunn’s test, *p* < 0.01, *r* = 0.60) and PPPD (median: 42.0 years, IQR: 8.0 years) groups (Dunn’s test, *p* < 0.0001, *r* = 0.74) (Figure 2 and Table 1).

**TABLE 1:** Clinical and demographic characteristics of those in the healthy control, persistent postural-perceptual dizziness, and unilateral vestibular hypofunction groups.

Variables	HC (*n* = 14)	PPPD (*n* = 27)	UVH (*n* = 12)	*P-value*
Sex, male/female	4/11	4/23	6/6	0.059
Age, years	46.0 (11.3)	42.0 (8.0)	65.5 (11.3)	< 0.0001****
Elliptical balance area (with eyes open), cm^2^	2.80 (2.58)	4.17 (5.39)	5.60 (4.68)	0.047*
Elliptical balance area (with eyes closed), cm^2^	4.36 (3.01)	8.39 (11.6)	8.97 (7.66)	< 0.001***
Romberg ratio on foam	1.73 (0.65)	1.87 (0.91)	2.14 (1.38)	0.193
Foam ratio	1.98 (0.76)	2.09 (1.01)	2.26 (1.75)	0.35
HADS (total score)	5.5 (7.5)	16.0 (8.0)	14.0 (11.0)	< 0.001***
HADS-A (anxiety)	4.0 (4.0)	8.0 (6.0)	5.5 (6.3)	< 0.01**
HADS-D (depression)	2.5 (4.3)	9.0 (4.0)	7.0 (5.5)	< 0.001***
Duration of disease, month	–	7.0 (22.0)	9.0 (10.8)	0.411
CP, %	–	9.8 (15.5)	52.4 (61.2)	< 0.0001****
DHI (total score)	–	48.0 (38.0)	33.0 (33.0)	0.048*
NPQ (total score)	–	36.0 (22.0)	21.0 (29.5)	0.027*
Upright posture/walking	–	10.0 (8.0)	8.5 (11.3)	0.217
Movement	–	10.0 (6.0)	9.0 (10.0)	0.121
Visual stimulation	–	14.0 (9.0)	6.0 (10.8)	< 0.01**

Values are reported as medians and interquartile ranges (IQRs), apart from the sex ratio reported in absolute values. Chi-square tests was performed for sex; Kruskal-Wallis test for age, elliptical balance area, Romberg ratio on foam, form ratio, and HADS; and Mann-Whitney U test for CP, DHI, and NPQ.

CP, canal paresis; DHI, dizziness handicap inventory; HADS, hospital anxiety and depression scale; HC, healthy controls; NPQ, Niigata PPPD questionnaire; PPPD, persistent postural-perceptual dizziness; UVH, unilateral vestibular hypofunction.

*Values indicate statistical significance. *p < 0.05; **p < 0.01; ***p < 0.001; ****p < 0.0001.

As shown in Table 1, the Kruskal−Wallis test demonstrated significant differences in the elliptical balance area (with eyes open/closed), HADS (total score), HADS-A (anxiety), and HADS-D (depression) among the three groups. The *post-hoc* Dann–Bonferroni test (Figure 2) revealed that the elliptical balance area (with eyes closed) of the HC group (median: 4.36, IQR: 3.01) was significantly smaller than that of the PPPD (median: 8.39, IQR: 11.6, *p* = 0.001, *r* = 0.55) and UVH (median: 8.97, IQR: 7.66, *p* = 0.002, *r* = 0.66) groups. The HADS and HADS-D of the HC group (HADS; median: 5.5, IQR: 7.5) (HADS-D; median: 2.5, IQR: 4.3) were significantly lower than those of the PPPD (HADS; median: 16.0, IQR: 8.0, *p* < 0.001, *r* = 0.64) (HADS-D; median: 9.0, IQR: 4.0, *p* < 0.001, *r* = 0.60) and UVH (HADS; median: 14.0, IQR: 11.0, *p* = 0.046, *r* = 0.48) (HADS-D; median: 7.0, IQR: 5.5, *p* < 0.05, *r* = 0.46) groups. The HADS-A of the HC group (median: 4.0, IQR: 4.0) was significantly lower than that of the PPPD group (median: 8.0, IQR: 6.0, *p* < 0.01, *r* = 0.54). No significant differences were observed between the PPPD and UVH groups in the HADS, HADS-A, and HADS-D.

The Mann–Whitney U test between the PPPD and UVH groups (Table 1) revealed that there was no significant difference in the duration of disease. The CP of the UVH group (median: 52.4, IQR: 61.2) was significantly greater than that of the PPPD group (median: 9.8, IQR: 15.5, *p* < 0.0001, *r* = 0.75), while the DHI (total score) (median: 48.0, IQR: 38.0) and NPQ (total score) (median: 36.0, IQR: 22.0) in the PPPD group were significantly greater than those in the UVH group (DHI; median: 33.0, IQR: 33.0, *p* = 0.048, *r* = 0.32) (NPQ; median: 21.0, IQR: 29.5, *p* = 0.027, *r* = 0.35). On the subscales of the NPQ, the visual stimulation score in the PPPD group (median: 14.0, IQR: 9.0) was significantly greater than that of the UVH group (median: 6.0, IQR: 10.8, *p* < 0.01, *r* = 0.49), while there were no significant differences between the two groups on the upright posture/walking score and movement score.

### Comparisons of the parameters of gaze stability in the pre-stimulus and post-stimulus states among the three groups

**FIGURE 3:**
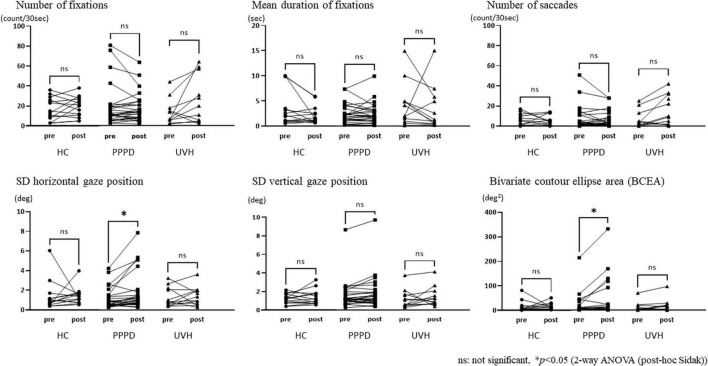
Comparisons of the parameters of gaze stability between pre- and post-stimuli states among the three groups. The standard deviation (SD) horizontal gaze position and bivariate contour ellipse area were significantly greater post-stimulus than pre-stimulus (SD horizontal gaze position; *p* = 0.050, *r* = 0.32) (bivariate contour ellipse area; *p* = 0.014, *r* = 0.38) in the persistent postural-perceptual dizziness group.

**FIGURE 4:**
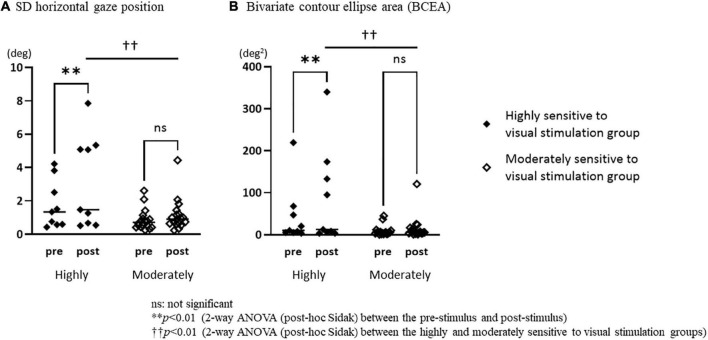
Comparisons of the standard deviation of the horizontal gaze position and bivariate contour ellipse area in the pre-stimulus and post-stimulus states between the highly and moderately sensitive to visual stimulation groups. **(A)** The standard deviation (SD) horizontal gaze position was significantly greater post-stimulus than pre-stimulus (*p* = 0.002, *r* = 0.69) in the highly sensitive to visual stimulation group, and the SD horizontal gaze position of the highly sensitive to visual stimulation group was significantly greater than that of the moderately sensitive to visual stimulation group (*p* = 0.003, *r* = 0.56) in the post-stimulus. **(B)** The bivariate contour ellipse area (BCEA) was significantly greater post-stimulus than pre-stimulus (*p* = 0.002, *r* = 0.68) in the highly sensitive to visual stimulation group, and the BCEA of the highly sensitive to visual stimulation group was significantly greater than that of the moderately sensitive to visual stimulation group (*p* = 0.006, *r* = 0.53) in the post-stimulus.

The repeated measures two-way ANOVA (factor group × factor pre-post) revealed that no significant interaction effects or main effects of factor group and factor pre-post were observed in the number of fixations, mean duration of fixations, number of saccades, and SD vertical gaze position (Table 2). In the SD horizontal gaze position and BCEA, no significant interaction effects or main effects of the factor group were found, while significant main effects of the factor pre-post were observed (SD horizontal gaze position; F = 4.99, *p* = 0.030) (BCEA; F = 5.53, *p* = 0.023) (Table 2). Figure 3 shows the results of the *post-hoc* Sidak test comparing the parameters of gaze stability before and after exposure to moving visual stimuli in the three groups. The SD horizontal gaze position and BCEA were significantly greater post-stimulus than pre-stimulus (SD horizontal gaze position; *p* = 0.050, *r* = 0.32) (BCEA; *p* = 0.014, *r* = 0.38) in the PPPD group. As a sensitivity analysis, the PPPD group was divided into two groups, one with visual stimulation scores of 19 or higher (highly sensitive to visual stimulation group) and the other with scores of less than 19 (moderately sensitive to visual stimulation group). The repeated measures two-way ANOVA (factor group × factor pre-post) was conducted again for SD horizontal gaze position and BCEA. Significant interaction effects (SD horizontal gaze position; F = 6.23, *p* = 0.020) (BCEA; F = 6.82, *p* = 0.015) and main effects of the factor group (SD horizontal gaze position; F = 7.11, *p* = 0.013) (BCEA; F = 5.81, *p* = 0.024) and factor pre-post (SD horizontal gaze position; F = 14.1, *p* < 0.001) (BCEA; F = 11.4, *p* < 0.01) were observed (Table 3). The *post-hoc* Sidak test revealed that the SD horizontal gaze position and BCEA were significantly greater at post-stimulus than pre-stimulus (SD horizontal gaze position; *p* = 0.002, *r* = 0.69) (BCEA; *p* = 0.002, *r* = 0.68), and the SD horizontal gaze position and BCEA were significantly greater in those who were highly sensitive to visual stimulation than in those who were moderately sensitive to visual stimulation (SD horizontal gaze position; *p* = 0.003, *r* = 0.56) (BCEA; *p* = 0.006, *r* = 0.53) (Figure 4).

**TABLE 2:** Comparisons of the parameters of gaze stability in the pre-stimulus and post-stimulus states among the three groups.

Variables	HC (*n* = 14)		PPPD (*n* = 27)		UVH (*n* = 12)		Interaction effect (*P-value*)	Main effects (*P-value*)	
	Pre	Post	Pre	Post	Pre	Post		Group	Pre-post
Number of fixations	14.0 (20.0)	20.5 (17.0)	13.0 (13.0)	15.0 (17.0)	10.5 (21.8)	24.0 (45.3)	0.57	0.621	0.755
(count/30 s)									
Mean duration of fixations	2.04 (4.10)	1.32 (1.97)	2.16 (2.35)	1.89 (2.15)	3.08 (3.84)	1.14 (5.15)	0.421	0.295	0.149
(s)									
Number of saccades,	3.5 (10.5)	3.5 (7.0)	2.0 (10.0)	3.0 (7.0)	3.5 (18.9)	9.5 (29.5)	0.989	0.065	0.769
(count/30 s)									
SD horizontal gaze position	0.91 (0.77)	1.25 (0.92)	0.78 (0.89)	1.00 (1.18)	0.86 (1.73)	1.14 (1.49)	0.301	0.925	0.030*
(degrees of visual angle)									
SD vertical gaze position	1.22 (0.94)	1.18 (1.08)	1.27 (1.00)	1.28 (1.16)	0.85 (1.20)	1.07 (1.22)	0.991	0.491	0.06
(degrees of visual angle)									
BCEA	5.4 (10.9)	11.8 (16.0)	5.9 (7.8)	6.8 (20.5)	5.4 (18.5)	9.5 (19.2)	0.194	0.505	0.023*
(square degrees of visual angle)									

Values are reported as medians and interquartile ranges. The repeated measures two-way analysis of variance was performed for all variables. BCEA, bivariate contour ellipse area; HC, healthy control; PPPD, persistent postural-perceptual dizziness; SD, standard deviation; UVH, unilateral vestibular hypofunction.

*Values indicate statistical significance. *p < 0.05.

**TABLE 3:** Comparisons of the standard deviation of the horizontal gaze position and bivariate contour ellipse area in the pre-stimulus and post-stimulus states between the highly and moderately sensitive to visual stimulation groups.

Variables	Highly sensitive to visual stimulation (*n* = 9)		Moderately sensitive to visual stimulation (*n* = 18)		Interaction effect	Main effects (*P-value*)	
	Pre	Post	Pre	Post	(*P-value*)	Group	Pre-post
SD horizontal gaze position	1.35 (2.58)	1.48 (4.61)	0.71 (0.56)	0.91 (0.65)	0.020*	0.013*	< 0.001***
(degrees of visual angle)							
BCEA	10.3 (52.5)	12.5 (148.6)	5.6 (5.5)	5.4 (10.5)	0.015*	0.024*	< 0.01**
(square degrees of visual angle)							

Values are reported as medians and interquartile ranges. The repeated measures two-way analysis of variance was performed for all variables. BCEA, bivariate contour ellipse area; SD, standard deviation.

*Values indicate statistical significance. *p < 0.05; **p < 0.01; ***p < 0.001.

### Parameters of gaze stability during exposure to moving visual stimuli

The repeated measures two-way ANOVA (factor group × factor visual stimuli) was performed to evaluate the SD horizontal gaze position and BCEA *during* exposure to moving visual stimuli, and no significant differences were noted in the interaction effects and main effects of the factor group, while significant main effects of the factor visual stimuli were observed (SD horizontal gaze position; F = 105.3, *p* < 0.0001) (BCEA; F = 10.8, *p* < 0.01) (Supplementary Table 3). Supplementary Figure 1 shows the results of the *post-hoc* Tukey test comparing the SD horizontal gaze position and BCEA *during* exposure to moving visual stimuli among the three moving visual stimuli. The SD horizontal gaze position was significantly higher during optokinetic stimulus by vertical stripes than during checkerboard and optic flow stimuli in all three groups. These tests demonstrated that horizontal eye movements were provoked during optokinetic stimulus even though the participants were instructed to stare at the center of the screen.

### Post/pre-bivariate contour ellipse area ratios in persistent postural-perceptual dizziness patients with or without antidepressant medication and exacerbation of dizziness sensation by exposure to moving visual stimuli in the gaze stability test

**FIGURE 5:**
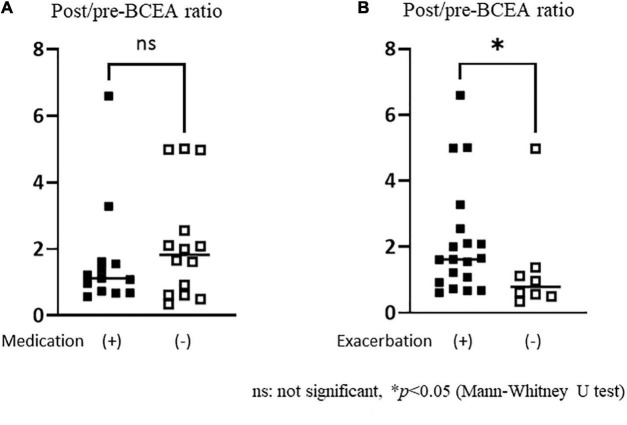
Comparisons of the post/pre-bivariate contour ellipse area (BCEA) ratios in persistent postural-perceptual dizziness patients with or without antidepressant medication and exacerbation of dizziness sensation. **(A)** There was no significant difference in the BCEA ratio between the groups with and without antidepressant medication. **(B)** The post/pre-BCEA ratio was significantly greater in the group with exacerbation of dizziness sensation [median: 1.63, IQR (interquartile range): 1.64] than in the group without (median: 0.79, IQR: 0.80, *p* = 0.029, *r* = 0.42).

There was no significant difference in the post/pre-BCEA ratio between the groups with and without antidepressant medication (Figure 5). Exacerbation of the dizziness sensation was observed in 19 out of 27 patients in the PPPD group, while no such sensation was reported in the HC and UVH groups. The post/pre-BCEA ratio was significantly higher in patients with exacerbation of the dizziness sensation (median: 1.63, IQR: 1.64) than in the those without exacerbation (median: 0.79, IQR: 0.80, *p* = 0.029, r = 0.42) in the PPPD group (Figure 5).

### Correlation between post/pre-bivariate contour ellipse area ratio and clinical parameters in the persistent postural-perceptual dizziness group

As shown in Table 4, there were no significant correlations between the post/pre-BCEA ratio and vestibular tests (elliptical balance area, Romberg ratio on foam, foam ratio, and CP) or clinical symptom scales (HADS, DHI, and NPQ).

**TABLE 4:** Correlation between post/pre-bivariate contour ellipse area ratio and clinical parameters in the persistent postural-perceptual dizziness group.

Variables	Spearman’s correlation coefficient r	95% confidence interval
Elliptical balance area (with eyes open), cm^2^	–0.082	−0.448 to 0.308
Elliptical balance area (with eyes closed), cm^2^	–0.027	−0.403 to 0.357
Romberg ratio on foam	–0.164	−0.512 to 0.230
Foam ratio	–0.154	−0.505 to 0.240
CP, %	–0.206	−0.556 to 0.206
HADS (total score)	0.241	−0.154 to 0.569
HADS-A (anxiety)	0.057	−0.331 to 0.427
HADS-D (depression)	0.357	−0.027 to 0.649
DHI (total score)	0.088	−0.302 to 0.453
NPQ (total score)	0.115	−0.277 to 0.474
Upright posture/walking	–0.019	−0.396 to 0.364
Movement	0.216	−0.179 to 0.551
Visual stimulation	0.118	−0.274 to 0.477

BCEA, bivariate contour ellipse area; CP, canal paresis; DHI, dizziness handicap inventory; HADS, hospital anxiety and depression scale; NPQ, Niigata PPPD questionnaire; PPPD, persistent postural-perceptual dizziness.

## Discussion

### Clinical and demographic characteristics of persistent postural-perceptual dizziness

In this study, age, elliptical balance area, and HADS (total score, anxiety, depression) were different among the HC, PPPD, and UVH groups. The *post-hoc* Dann–Bonferroni test demonstrated that patients with PPPD were significantly younger than those with UVH and had a higher HADS score and broader elliptical balance area (with eyes closed) in posturography compared with the HCs. These clinical and demographic characteristics, such as a relatively young distribution with anxious/depressive background observed in the present PPPD group, were consistent with those in previous studies (Kim et al., 2020; Bittar and von Sohsten Lins, 2015). The results of posturography in this study were consistent with those in previous reports (Sohsten et al., 2016; McCaslin et al., 2022), showing significant differences in the eyes-closed condition but not in the eyes-open condition compared with HCs.

Patients with PPPD had precipitating vestibular conditions, such as an acute attack of peripheral vestibular vertigo, Meniere’s disease, and vestibular neuritis; however, CP% was within normal range and remained significantly lower than that of patients with UVH. Nonetheless, the DHI scores and NPQ scores of patients with PPPD were significantly higher than those of patients with UVH, indicating that patients with PPPD had more severe subjective handicaps, even with normal vestibular function; this is consistent with a previous report (Kitazawa et al., 2021). On the NPQ subscale, the PPPD group scored significantly higher than the UVH group on visual stimulation, indicating that visual exacerbation is specifically observed in the PPPD group, as in previous reports (Yagi et al., 2019).

### Changes in gaze stability before and after exposure to moving visual stimuli

There were no significant differences in each parameter of gaze stability among the three groups at pre-stimulus. It is suggested that the basic gaze stability in those with PPPD did not largely differ between HC and UVH groups. Nonetheless, the BCEA at post-moving visual stimuli was significantly higher than that at pre-moving visual stimuli only in the PPPD group. The formula for calculating BCEA incorporates the value of the SD horizontal gaze position (see the “measurement of gaze stability” subsection of the Methods). Since the SD horizontal gaze position increased significantly in the PPPD group after exposure to moving visual stimuli, the BCEA also increased as a result of the increased SD horizontal gaze position, that is, a large dispersion of gazing position in the horizontal direction. There were no significant post-stimulation differences in the SD horizontal gaze position and BCEA among the three groups, and the effect sizes were moderate; however, it is suggested that gaze instability, such as an increased SD horizontal gaze position and the BCEA induced by exposure to moving visual stimuli, would be a potential tendency of eye movements in PPPD.

### Mechanisms of symptoms exacerbation by visual stimuli in persistent postural-perceptual dizziness

An increase in BCEA has been reported in individuals with anxiety and aging (Laretzaki et al., 2011; Altemir et al., 2021). Altemir et al. (2021) investigated changes in the BCEA by age in healthy subjects and reported that gaze stability gradually worsened from the fifth decade of life. In this study, patients with PPPD showed a relatively young distribution, suggesting that the increase in BCEA observed in the PPPD group after exposure to moving visual stimuli was not due to aging. Regarding the possible increase in BCEA by anxiety, there were no significant differences in HADS-A, a scale of anxiety, between the PPPD and UVH groups. Taken together, the increase in BCEA in the PPPD group was unlikely to have been caused by psychological and aging factors.

We used three different moving visual stimuli that mimic symptom exacerbators in daily life. Among these, the optokinetic stimulus had a greater impact on the SD horizontal gaze position *during* exposure to moving visual stimuli than the other two stimuli, irrespective of the groups. That is, the horizontal eye movements were provoked *during* optokinetic stimuli for all three groups, even though the participants were instructed to stare at the center of the screen. However, after exposure to moving visual stimuli, an increase in the SD horizontal gaze position and BCEA were observed only in the PPPD group. It is suggested that the gaze instability induced by the moving visual stimuli is sustained in patients with PPPD even several moments after the stimulation. Since the post/pre-BCEA ratio in the PPPD group was significantly higher in those who showed exacerbation of dizziness sensation after moving visual stimuli than in those who did not, it is suggested that the sustained gaze instability induced by moving visual stimuli could be causing the exacerbation of dizziness symptoms in PPPD. This is consistent with the results of a sensitivity analysis, in which those who were highly sensitive to visual stimulation, who had severe symptom exacerbation due to visual stimuli in daily life, showed significant gaze instability compared with the moderately sensitive to visual stimulation group.

Although sedative medications are known to affect eye-gaze patterns, such as increasing saccade numbers and shorter fixation duration (Jonassen et al., 2015), the post/pre-BCEA ratio was not different between those who received medication and those who did not. It is suggested that BCEA enlargement may be linked to symptom exacerbation and not to medication-induced effects in the PPPD group.

### Limitations

There are several limitations to this study. First, the eye-tracking device used in this study had a low sampling rate of 60 Hz, which made the correct identification of saccades or abnormal eye movements difficult, and micro-saccadic activity could not be detected. Future studies using devices with larger sampling rates are needed. Second, whether gaze instability is a cause or result of symptom exacerbation remains unclear. Third, since psychological states were evaluated using the HADS as a routine test battery for dizzy patients, and those immediately after the moving visual stimulation were not evaluated, the relevance of the psychological factors might have been underestimated. Fourth, the sample size in this study is small. When comparing the gaze stability test before and after visual stimulation among the three groups, the power of the *post-hoc* test is 0.57, and ideally, at least 15 cases in each group are required. In addition, due to the small sample size, statistical analyses of precipitants and comorbidities were not performed. Moreover, duration for treatments at the time of the gaze stability test could not be tested due to the difficulty of investigating the detailed data. The possibility remains that these factors might have affected the results of this study.

## Conclusion

Patients with PPPD were more likely to exhibit gaze instability after exposure to moving visual stimuli, which potentially exacerbated vestibular symptoms. This phenomenon may help elucidate the neural mechanisms of visual exacerbation in PPPD.

## Data availability statement

The data supporting this study’s findings are available from the corresponding author upon reasonable request.

## Ethics statement

This study was approved by the Institutional Review Board of Niigata University Medical and Dental Hospital (Niigata City, Japan) (#2020-0242). All procedures performed in this study were in accordance with the ethical standards of the institutional and/or National Research Committee and the 1964 Helsinki Declaration. Informed consent was obtained from all the participants at the time of inclusion in the study, authorizing the anonymous use of data for further studies.

## Author contributions

CY and AH contributed to the conception and design of the manuscript. YM, TY, SO, SI, KT, KI, YS, and HI made substantial contributions to the conception of the work and were responsible for data collection. CY wrote the manuscript. All authors contributed to the manuscript revision, read, and approved the submitted version.
