# Supplementary material for: Gaze instability after exposure to moving visual stimuli in patients with persistent postural-perceptual dizziness
Source: Front Hum Neurosci. 2022 Nov 25;16:1056556. doi: 10.3389/fnhum.2022.1056556 (PMC9733075; doi:10.3389/fnhum.2022.1056556)
Supplement: Supplementary file 1 [file Data_Sheet_1.PDF]

frontiers

Supplementary Material

Supplemental Table 1. Precipitating conditions of patients with persistent postural-perceptual dizziness (PPPD) and unilateral vestibular hypofunction (UVH) (n)

PPPD	n = 27	UVH	n = 12
Acute attack of peripheral vestibular vertigo	n = 9	Sudden deafness with vertigo	n = 3
BPPV	n = 8	No specific precipitants	n = 3
Chronic anxiety disorders	n = 3	Vestibular neuritis	n = 2
Meniere's disease	n = 2	Ramsay Hunt syndrome	n = 2
No specific precipitants	n = 2	Acute attack of peripheral vestibular vertigo	n = 1
Vestibular neuritis	n = 1	Postoperative status of acoustic tumor	n = 1
Sudden deafness with vertigo	n = 1		
Cerebellar infarction	n = 1		

Abbreviations: BPPV, benign paroxysmal positional vertigo

Supplemental Table 2. Details of the Tobii I-VT algorithm

Gap fill-in (interpolation)	Enabled<br>Max. gap length: 75 ms
Eye selection	Average
Noise reduction	Moving Median<br>Window size: 5 samples
Velocity calculator	Window length 20 ms
I-VT fixation classifier	Threshold 30 °/s
Merge adjacent fixations	Enabled<br>Max. time between fixations: 75 ms<br>Max. angle between fixations: 0.5 °
Discard short fixations	Enabled<br>Minimum fixation duration: 60 ms

Supplemental Table 3. Comparisons of the standard deviation of the horizontal gaze position and bivariate contour ellipse area *during* exposure to moving visual stimuli for each stimulus in the three groups

Valuable	Group	(i) Checkerboard	(ii) Stripes	(iii) Optic flow	Interaction effect (*p*-value)	Main effects (*p*-value)	
						Group	Visual stimuli
SD horizontal gaze position (degrees of visual angle)	HC	1.30 (1.09)	3.58 (3.66)	1.07 (1.47)	0.245	0.912	<0.0001****
	PPPD	1.22 (0.63)	3.37 (2.10)	1.22 (1.05)			
	UVH	0.99 (0.83)	3.12 (1.69)	1.20 (1.25)			
BCEA (square degrees of visual angle)	HC	10.5 (18.3)	33.9 (61.1)	12.1 (37.0)	0.363	0.641	<0.01**
	PPPD	13.2 (17.0)	48.4 (47.5)	11.8 (21.8)			
	UVH	6.2 (12.8)	30.0 (49.2)	14.6 (35.0)			

Values are reported as medians and interquartile ranges. The repeated measures two-way analysis of variance was performed for all variables. Abbreviations: BCEA, bivariate contour ellipse area; SD, standard deviation

*Values indicate statistical significance. **p<0.01; ****p<0.0001

Supplemental Figure 1. Comparisons of the standard deviation of the horizontal gaze position and bivariate contour ellipse area *during* exposure to moving visual stimuli among the three moving visual stimuli

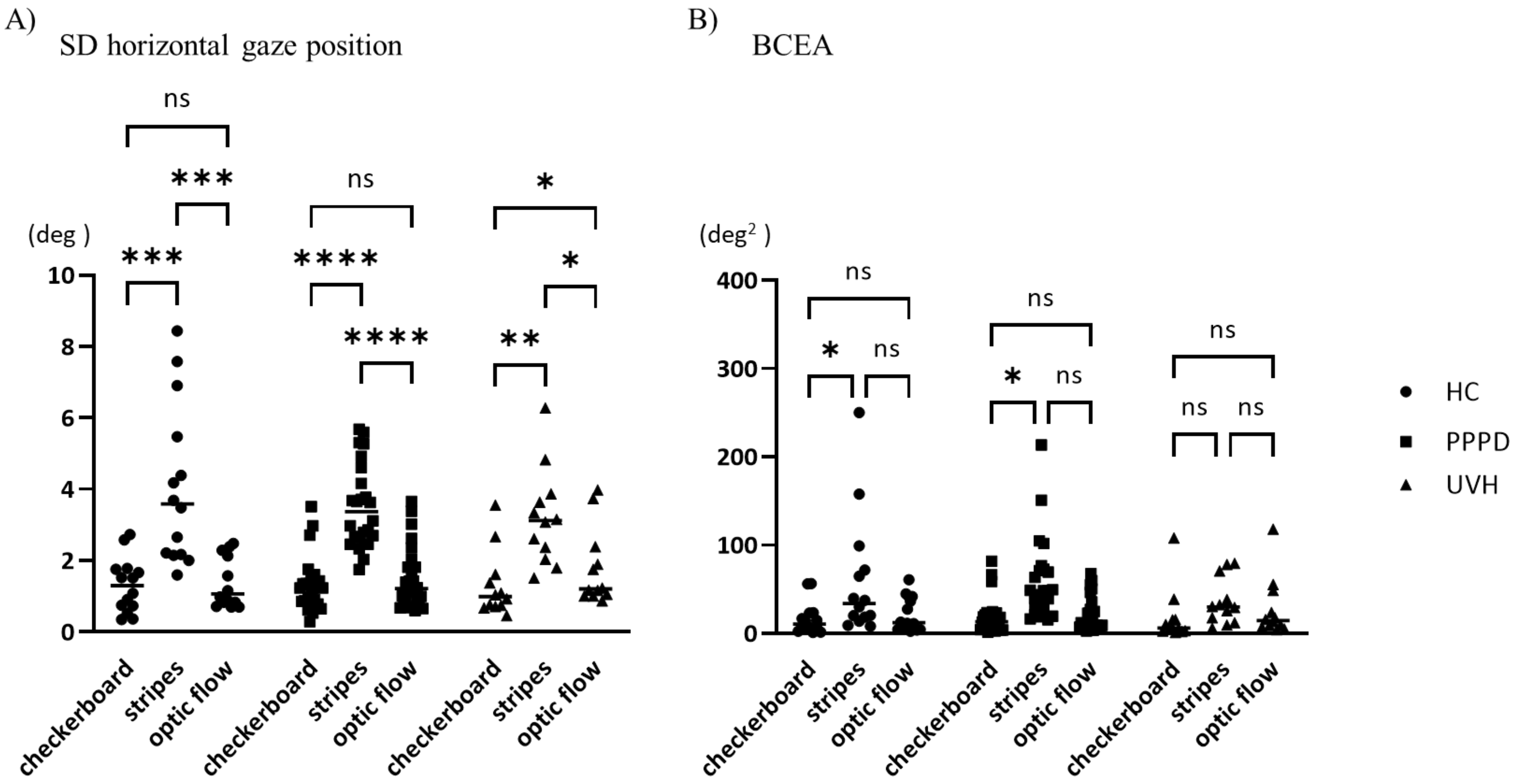

*p<0.05, **p<0.01, ***p<0.001, ****p<0.0001(2-way ANOVA (post-hoc Tukey))
